# Evaluation of multinodular goiter and primary hyperparathyroidism leads to a diagnosis of AL amyloidosis

**DOI:** 10.1186/s13044-022-00125-5

**Published:** 2022-04-20

**Authors:** Chandani Patel Chavez, Maria del Mar Morales Hernandez, Jesse Kresak, Whitney W. Woodmansee

**Affiliations:** 1 Division of Endocrinology, Diabetes and Metabolism, University of Florida, Gainesville, FL USA; 2 Department of Pathology, Immunology and Laboratory Medicine, College of Medicine, University of Florida, Gainesville, FL USA

**Keywords:** Amyloid goiter, Amyloid, AL amyloidosis, Amyloid light chain

## Abstract

**Background:**

Amyloid goiter, defined as excess amyloid within the thyroid gland in such quantities that it produces a clinically apparent goiter, is a very rare manifestation of systemic amyloidosis with cases commonly seen in the setting of Amyloid A (AA) amyloidosis. Amyloid goiter as the primary clinical manifestation secondary to Amyloid light chain (AL) amyloidosis is very rare. We present a case of AL amyloidosis with initial manifestation as goiter with amyloid deposition in the thyroid and the parathyroid gland.

**Case Presentation:**

A 73 year old male presented with goiter and compressive symptoms of dysphagia and hoarseness. Laboratory workup revealed normal thyroid function, nephrotic range proteinuria, elevated serum calcium level with an elevated parathyroid hormone level (PTH) consistent with primary hyperparathyroidism. Thyroid ultrasound showed an asymmetric goiter with three dominant nodules. Cervical computed tomography revealed a goiter with substernal extension and deviation of the trachea. Fine needle aspiration was unsatisfactory. There was also evidence of osteoporosis and hypercalciuria with negative Sestamibi scan for parathyroid adenoma. The patient underwent a total thyroidectomy and one gland parathyroidectomy. Pathology revealed benign thyroid parenchyma with diffuse amyloid deposition in the thyroid and parathyroid gland that stained apple green birefringence under polarized light on Congo Red stain. Immunochemical staining detected AL amyloid deposition of the lambda type. Bone marrow biopsy revealed an excess monoclonal lambda light chain of plasma cells consistent with a diagnosis of AL amyloidosis secondary to multiple myeloma affecting the kidney, thyroid, parathyroid gland, and heart. He was treated with 4 cycles of chemotherapy with a decrease in the M spike and light chains with a plan to pursue a bone marrow transplant.

**Conclusion:**

Amyloid goiter as the primary clinical manifestation secondary to AL amyloidosis with deposition in the thyroid and parathyroid gland is rare. The top differential for amyloid deposits in the thyroid includes systemic amyloidosis or medullary thyroid carcinoma. The definitive diagnosis lies in the histopathology of the thyroid tissue. To diagnose systemic amyloidosis as the etiology for a goiter, a solid understanding of the causes of systemic amyloidosis coupled with a thorough evaluation of the patient’s history and laboratory data is necessary.

## Background

Amyloidosis is a heterogenous group of diseases in which extracellular deposits of insoluble beta pleated proteins known as amyloid build up in vital organs, commonly the heart, kidneys, and liver [1]. The most common cause of an amyloid disorder in the developed world is AL amyloidosis (previously known as primary amyloidosis) wherein the amyloid consists of pathologic immunoglobulin light chains in the setting of monoclonal plasma cell dyscrasias (i.e., multiple myeloma) or lymphoproliferative disorders [2]. Alternatively, the most common cause of amyloidosis in the developing world is AA amyloidosis (previously known as secondary amyloidosis) wherein the amyloid deposits consists of the acute phase reactant, amyloid A, present in chronic inflammatory conditions such as Familial Mediterranean Fever (FMF), rheumatoid arthritis, inflammatory bowel disease, chronic infections, or neoplasms [3].

Amyloid deposits in the thyroid were first discovered by Von Rokitansky in 1855 based on autopsy results but it was clinically recognized in 1858 by Beckman. The term amyloid goiter was officially adopted in 1904 when Von Eiselberg defined the presence of amyloid in the thyroid gland producing significant clinical enlargement of the gland and is a rare manifestation of systemic amyloidosis [4].

We describe a patient who presented with an 18 month history of a goiter with compressive symptoms and concurrent primary hyperparathyroidism who underwent a total thyroidectomy. The goiter and parathyroid gland were unexpectedly found to be infiltrated with AL amyloid deposition of the lambda light chain subtype. Subsequent workup revealed systemic AL amyloidosis secondary to multiple myeloma with involvement not only of the thyroid but also of the parathyroid gland, heart and kidneys.

## Case presentation

A 73 year old male was evaluated by endocrinology in October of 2020 for a rapidly enlarging (18 month) right-sided non-tender multinodular goiter and hypercalcemia. He reported associated dysphagia with solid foods and a progressively hoarse voice. Also endorsed multiple episodes of kidney stone, two fragility fractures and short-term memory problems. Denied dyspnea or orthopnea. There was no history of radiation to the head or neck. Family history significant for a goiter in his son with no history of thyroid cancer. He had a medical history significant for a pituitary adenoma resected in 1998, left adrenal adenoma, hypertension, nephrolithiasis, and obesity status post Roux-en-Y bypass. The patient reported a history of a non-functioning pituitary adenoma however preoperative findings and pathology were unavailable. He was not on any pituitary hormonal replacement therapy at time of diagnosis, and reported he was treated briefly post-operatively with growth hormone. The most recent MRI pituitary showed an atrophic pituitary gland. At time of consultation, the patient reported a history of a stable non-functioning adrenal adenoma.

**Fig. 1:**
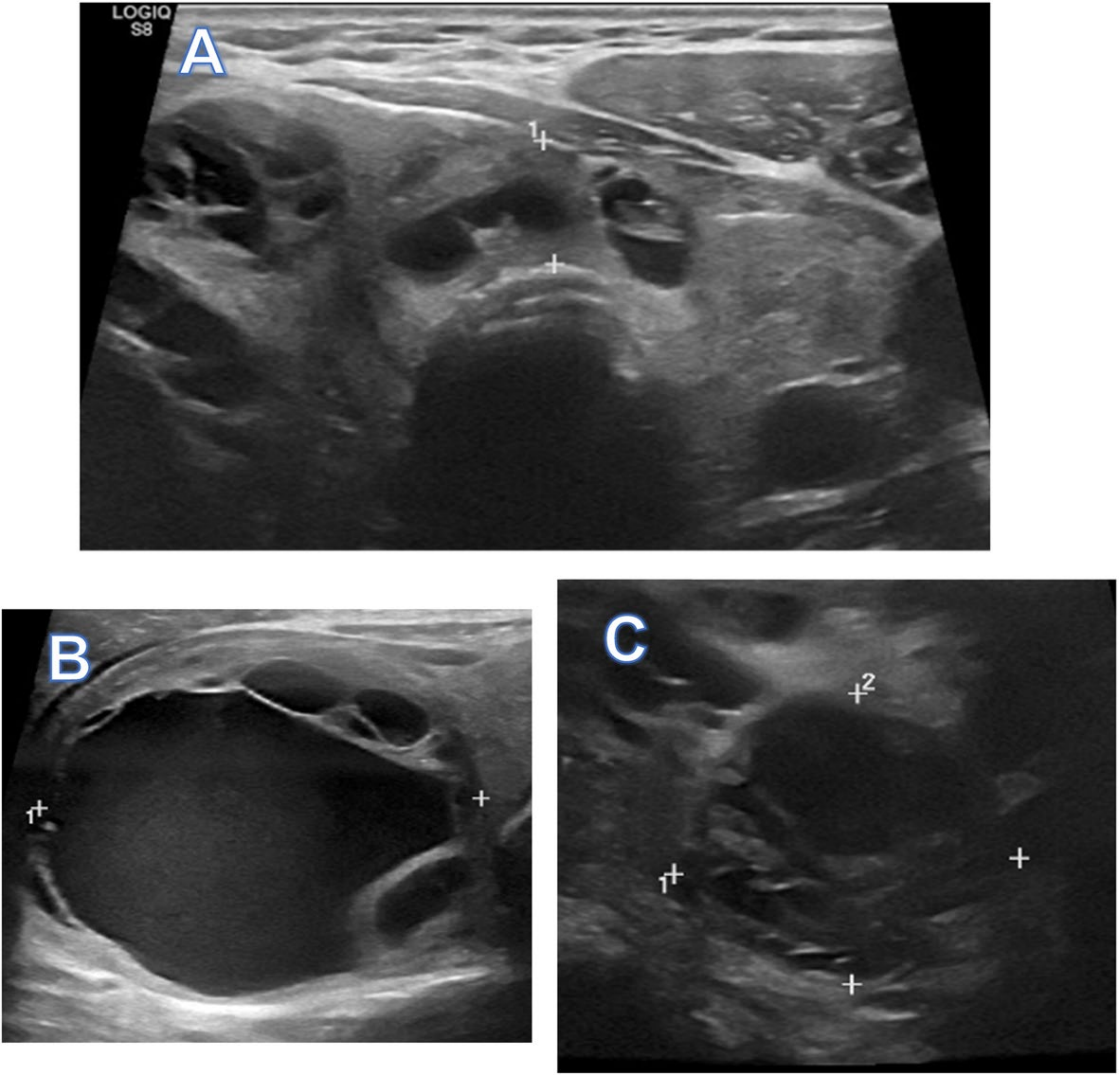
Transverse view ultrasound. **A** Asymmetrically enlarged, heterogenous thyroid. **B** Right mid thyroid nodule with mixed cystic and solid components measured 3.9 × 4.3 × 4.9 cm in size. **C** Right inferior thyroid nodule with mixed cystic and solid components measured 2.5 × 2.2 × 2.9 cm in size

**Fig. 2:**
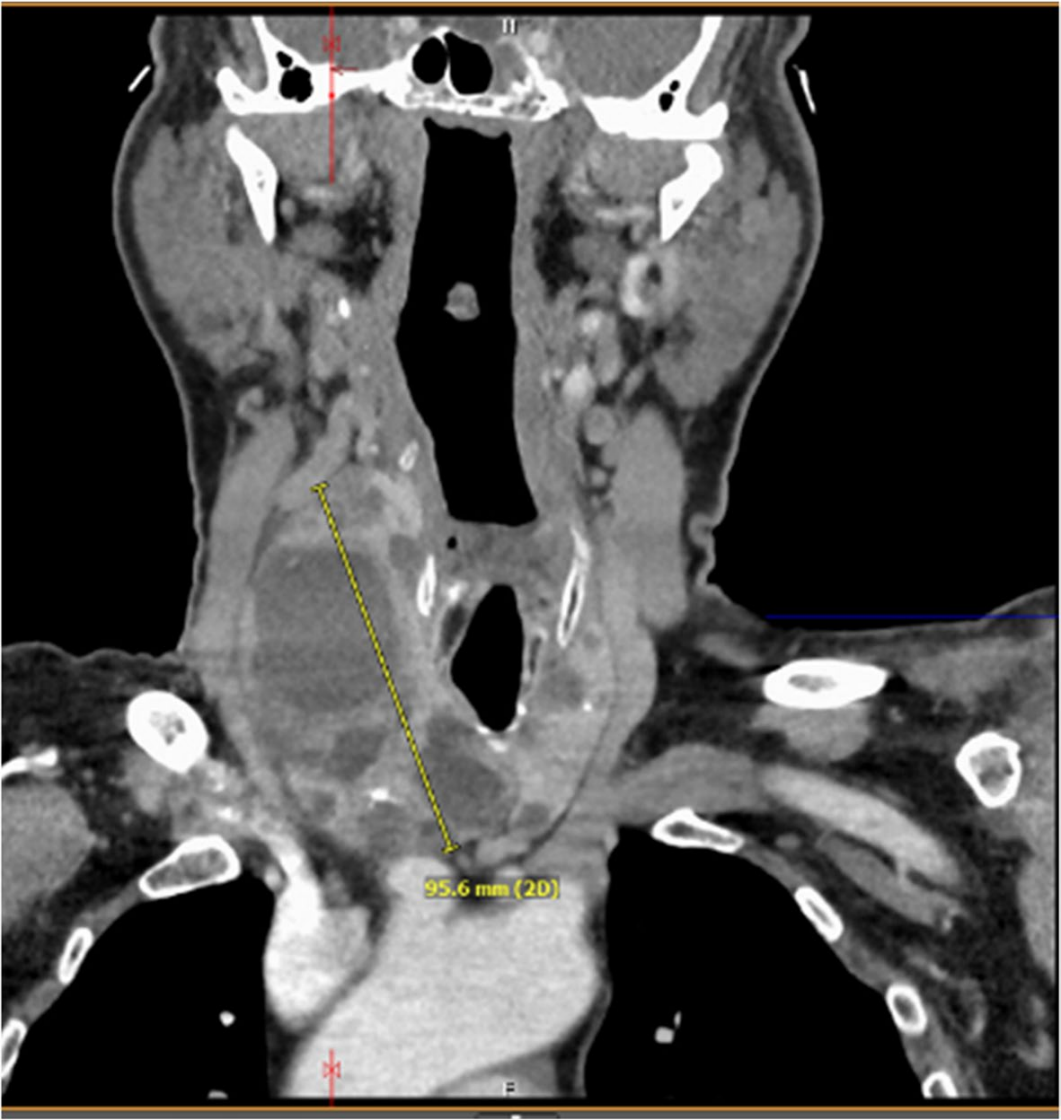
Cervical computed tomography. Enlarged right thyroid lobe (AP, W, CC) of 5.5 × 6.0 × 10.0 cm compared to the left lobe with multiple cystic and complex nodules. Extends into the upper substernal region displacing the great vessels, 60% compression of the trachea, with 1.8 cm leftward deviation of the trachea

Thyroid studies revealed TSH 2.94 mIU/L (ref 0.40 – 4.50 mIU/L), Free T4 0.8 ng/dL (ref 0.8 -1.8 ng/dL), Free T3 3.0 pg/mL (ref 2.3–4.2 pg/mL), thyroglobulin antibody < 1.0 IU/mL (ref < 1.0 IU/mL), thyroid peroxidase antibodies < 1.0 IU/mL (ref < 9 IU/mL). A thyroid ultrasound revealed a markedly heterogeneous goiter. The right lobe measured 4.5 × 4.8 × 10.0 cm (anteroposterior × width × length) and the left lobe measured 2.5 × 2.1 × 5.7 cm (Fig. 1A). “There were three dominant nodules (TIRADS 3) noted in the right thyroid lobe including a right mid thyroid nodule with mixed cystic and solid components measured 3.9 × 4.3 × 4.9 cm in size (Fig. 1B); right inferior thyroid nodule with mixed cystic and solid components measured 2.5 × 2.2 × 2.9 cm in size (Fig. 1C); right inferior thyroid nodule with mixed cystic and solid components measured 2.6 × 2.6 × 2.0 cm in size. Cervical computed tomography (CT) revealed a markedly enlarged right thyroid lobe (AP, W, CC) of 5.5 × 6.0 × 10.0 cm compared to the left lobe with multiple cystic and complex nodules (Fig. 2). It extended into the upper substernal region displacing the great vessels and there was 60% compression of the trachea, with 1.8 cm leftward deviation of the trachea. Fine needle aspiration (FNA) of the three dominant nodules in the right were unsatisfactory (Bethesda Category I), only showing a few macrophages and inflammatory cells.

In addition to the goiter, he was also found to have PTH mediated hypercalcemia. Initial laboratory investigations revealed a hemoglobin 13.3 g/dL (ref 13 – 16.5 mg/dL), a calcium 9.9 mg/dL (ref 8.6–10.3 mg/dL), albumin 3.0 g/dL (ref 3.6–5.1 g/dL) with corrected calcium of 10.7 mg/dL, total protein 5.5 g/dL (ref 6.1–8.1 g/dL), creatinine 1.10 mg/dL (ref 0.7 – 1.18 mg/dL), eGFR 67 mL/min, ionized calcium 5.9 mg/dL (ref 4.8–5.6 mg/dL), intact parathyroid hormone level 147 pg/mL (ref 14–64 pg/mL), phosphate 2.8 mg/dL (ref 2.1–4.3 mg/dL), 25-hydroxy vitamin D 41 ng/mL, 1,25-Dihydroxyvitamin D 107 ng/mL (ref 18–72 ng/mL), 24 urine calcium 449 mg/24 h (ref 5–300 mg/24 h). A Sestamibi scan found no scintigraphic evidence of a parathyroid adenoma. A bone densitometry scan revealed osteoporosis in the femoral neck (T score, –3.8) and distal radius (T score, -2.8). Given the multinodular goiter was causing compressive symptoms and extending substernally into the chest, a total thyroidectomy was performed. He was also diagnosed with primary hyperparathyroidism and given the history of nephrolithiasis, hypercalciuria and osteoporosis, a parathyroidectomy was performed simultaneously.

**Fig. 3:**
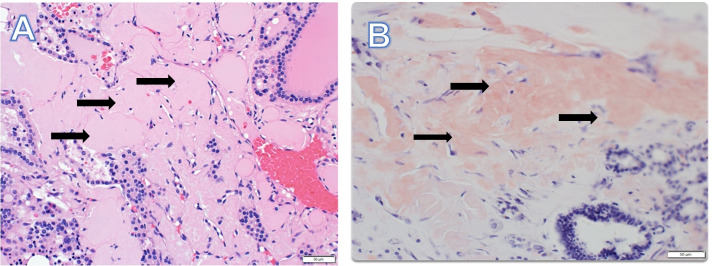
Black arrows point to extensive interstitial and nodular deposition of amyloid which appears as amorphous eosinophilic material on H&E stain (**A**) and highlights a salmon-color on Congo Red stain (**B**)

**Fig. 4:**
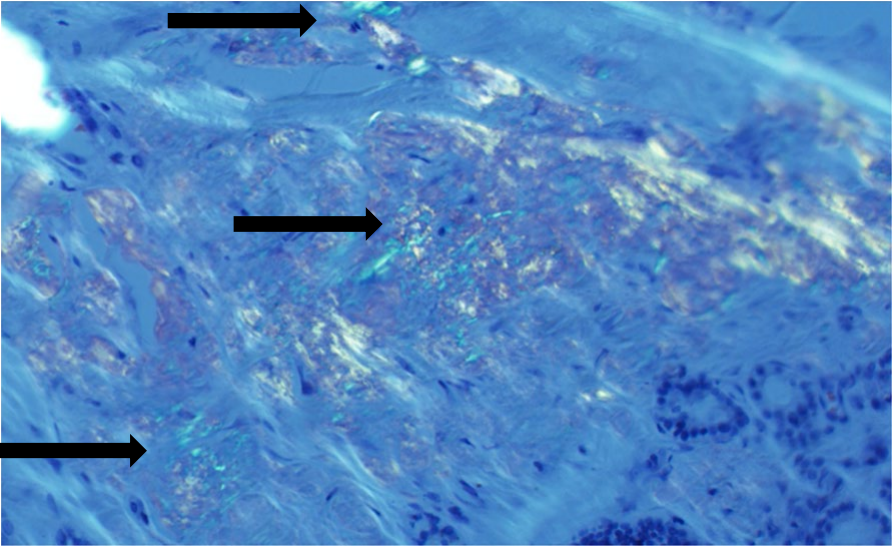
Amyloid is histologically confirmed by the typical “apple-green” birefringence (black arrows) of the amyloid protein as seen under cross-polarized light of a Congo Red stain

The thyroid specimen consisted of a 8.0 × 7.0 × 3.5 cm right lobe, 2.5 × 1.0 × 1.0 cm isthmus and 1.5 × 5.0 × 2.0 cm left lobe and weighed 92 g. There were multiple well-circumscribed nodules ranging from 1.2 cm to 4.0 cm in size, with few containing a gelatinous surface and one with areas of hemorrhage. Pathology revealed benign thyroid parenchyma with diffuse amyloid deposition (Fig. 3A and B). It was difficult to locate the parathyroid glands with multiple specimens submitted to pathology. Final pathology revealed only one right superior parathyroid gland was removed, with amyloid deposition in the parathyroid tissue. Amyloid was confirmed on Congo red staining with green birefringence under polarized light (Fig. 4) and stained negative for calcitonin. Liquid chromatography tandem mass spectrometry of tissue detected a peptide profile consistent with AL amyloid deposition of the lambda type. Immunofixation serum electrophoresis demonstrated an IgG lambda monoclonal immunoglobulin. Bone marrow biopsy and aspirate showed 15 to 20% of monoclonal lambda light chain plasma cells by flow cytometry analysis. There were no amyloid protein deposits. A skeletal survey was negative for any bone lytic lesions. A 24 urine protein collection showed nephrotic range proteinuria (7140 mg/g creatinine). Cardiac workup showed low voltage on ECG and echocardiogram with left ventricular hypertrophy with bright myocardial pattern suggestive of mild cardiac amyloidosis.

A diagnosis of AL amyloidosis secondary to multiple myeloma affecting the kidney, thyroid, parathyroid gland and likely heart was made. He was started on chemotherapy with Bortezomib, Lenalidomide and Dexamethasone and completed 4 cycles. The M spike decreased from 0.81 g/dL to 0.23 g/dL and light chains trended down from 7.41 mg/dL to 5.51 mg/dl (0.57—2.63 mg/dL). Oncology plans to pursue a bone marrow transplant. Given the history of pituitary adenoma and primary hyperparathyroidism, he will also be seeing a medical geneticist to evaluate for multiple endocrine neoplasia type 1 syndrome (MEN).

## Discussion /conclusion

Amyloid deposits can be seen in the thyroid commonly in systemic amyloidosis, estimated between 20 and 50% in AL amyloidosis and up to 80% in AA amyloidosis [5]. On the other hand, a goiter from a large quantity of amyloid deposition known as amyloid goiter is very rare. It occurs in only 0.04% of patients with systemic amyloidosis [6]. The majority of cases are associated with AA amyloidosis from chronic inflammatory states. AL amyloidosis as the etiology of amyloid goiter is even more rare and predominantly reported as case reports. A systematic review of 30 cases revealed AA amyloidosis, specifically FMF, as the most common cause of amyloid goiter [7]. The mean age was 44 and male predominant [7]. Otherwise, the differential for amyloid deposits in the thyroid is quite narrow. Amyloid deposition can be seen in 30–50% of patients with medullary thyroid carcinoma (MTC) [8] with amyloid consisting of calcitonin or pre-procalcitonin and very rarely in microfollicular thyroid cancer [9] and Riedel’s thyroiditis [10]. It is crucial to rule out medullary thyroid carcinoma as the etiology especially in this patient given there is concern for MEN. Medullary thyroid carcinoma is a common manifestation associated with MEN2, a more likely possibility in this patient but there are a few rare case reports of MTC occurring in MEN1 [11]. Amyloid deposit in the parathyroid glands is also clinically rare and can go easily undetected in patients with systemic amyloidosis [12]. An autopsy series of 30 parathyroid glands of 8 different patients found amyloid deposition in all the individuals suggesting parathyroid amyloid deposition can be more common than clinically established [13]. Amyloid deposits were found in this patient’s parathyroid gland but is unlikely the cause of his primary hyperparathyroidism given the patient continues to have persistent PTH elevation after his parathyroidectomy and amyloid infiltration of the parathyroid is commonly associated with hypoparathyroidism.

Amyloid goiter usually presents with a rapidly growing gland with compressive symptoms such as dyspnea, dysphagia and dysphonia [14]. Our patient presented with both enlarging goiter and compressive symptoms as described in other case reports. Even though must amyloid goiters are rapid growing, Orrego et al. reported a case of a slow growing goiter Bethesda category III as per cytology with large amyloid deposit on congo staining in the surgical pathology [14]. Generally, patients with amyloid goiter are euthyroid but some case reports described association with hypothyroidism or hyperthyroidism [14, 15]. For example, Mohammed et al. described a case of primary localized amyloidosis in the thyroid and hypothyroidism [16, 17].

Evaluation of goiter usually involves imaging modalities like ultrasonography, CT scans or magnetic resonance imaging. Imaging patterns vary according to the proportion of adipose tissue in relation to the amyloid [18, 19]. Ultrasound can show a diffusely enlarged heterogeneous thyroid gland, with focal deposits of amyloid detected as complex or hypoechoic masses [19]. The diagnosis of amyloid goiter is challenging given the definitive diagnosis is made by histopathology of resected thyroid tissue, therefore a high clinical suspicion is crucial for diagnosis. The role of FNA as a diagnostic tool remains inconclusive in the literature. Typical findings on hematoxylin and eosin-stained FNA smears can include irregular fragments of pink-stained amorphous material [6, 7]. If the cellular smear is stained with Congo-red and examined under polarized light, there is the characteristic positively stained amyloid fibrils with apple green birefringence [7]. Otherwise, in 10 to 40% of cases, FNA will show atypical follicular cells or inconclusive findings as seen in our patient [7]. Definitive diagnosis is based on histologic evaluation of resected thyroid tissue where amyloid is present extracellularly as amorphous, eosinophilic proteinaceous material under the light microscope [7]. The amyloid material commonly infiltrates and distorts the normal tissue architecture [7]. Other histologic findings include fatty metaplasia and squamous metaplasia [20]. The Congo-Red stain will reveal amyloid deposits as apple green birefringence under polarized light and is deemed the pathognomonic feature [21]. Special immunohistochemical staining may help differentiate the constituents of the amyloid deposits as amyloid A or amyloid AL [22]. Medullary thyroid carcinoma can be ruled out if the resected tissue stains are negative for calcitonin, chromogranin A or carcinoembryonic antigen (CEA) [23].

Treatment of amyloid goiter requires thyroidectomy for management of compressive symptoms. Patients with systemic amyloidosis will require management of the underlying primary process and like in our case, chemotherapy was required. Law et al. reported one case of amyloid goiter secondary to systemic amyloidosis treated with a stem cell transplantation and high dose dexamethasone therapy [24]. The patient’s goiter shrank from 50 to 30 g with stability in size of the goiter 6 years after treatment [24].

In this patient with a history of a pituitary adenoma, primary hyperparathyroidism and left adrenal nodule, a diagnosis of multiple endocrine neoplasia syndrome was high on the differential. Although MTC is a common manifestation associated with MEN2, a more likely possibility in this patient, it has occurred rarely in MEN1. Therefore, MTC was on the differential for this patient who presented with a multinodular goiter. Amyloid deposits can be seen in medullary thyroid carcinoma and it was vital to rule this out with the negative calcitonin stain. As evidenced in this case and other case reports, we cannot confidently rely on fine needle aspiration and histopathology of the resected thyroid tissue was needed for the definitive diagnosis. This unique, complicated endocrine case highlights the importance of understanding the causes of amyloid goiter and performing a thorough evaluation of a patient’s history and laboratory data to arrive at a diagnosis of AL amyloidosis.

## Data Availability

The datasets used during the current study are available from the corresponding author on reasonable request.

## Abbreviations

AA: Amyloid A
AL: Amyloid light chain
PTH: Parathyroid hormone
FMF: Familial Mediterranean Fever
CT: Cervical computed tomography
FNA: Fine needle aspiration
MEN: Multiple endocrine neoplasm
MTC: Medullary Thyroid Carcinoma
CEA: Carcinoembryonic antigen
